# Influence of an acetate- and a lactate-based balanced infusion solution on acid base physiology and hemodynamics: an observational pilot study

**DOI:** 10.1186/2047-783X-17-21

**Published:** 2012-07-06

**Authors:** Klaus F Hofmann-Kiefer, Daniel Chappell, Tobias Kammerer, Matthias Jacob, Michaela Paptistella, Peter Conzen, Markus Rehm

**Affiliations:** 1 Clinic of Anesthesiology, Ludwig-Maximilians University, City of Munich, Germany

**Keywords:** Acetate, Lactate, Balanced infusion solution, Acid–base balance, Hemodynamic stability

## Abstract

**Background:**

The current pilot study compares the impact of an intravenous infusion of Ringer’s lactate to an acetate-based solution with regard to acid–base balance. The study design included the variables of the Stewart approach and focused on the effective strong ion difference. Because adverse hemodynamic effects have been reported when using acetate buffered solutions in hemodialysis, hemodynamics were also evaluated.

**Methods:**

Twenty-four women who had undergone abdominal gynecologic surgery and who had received either Ringer’s lactate (Strong Ion Difference 28 mmol/L; n = 12) or an acetate-based solution (Strong Ion Difference 36.8 mmol/L; n = 12) according to an established clinical protocol and its precursor were included in the investigation. After induction of general anesthesia, a set of acid–base variables, hemodynamic values and serum electrolytes was measured three times during the next 120 minutes.

**Results:**

Patients received a mean dose of 4,054 ± 450 ml of either one or the other of the solutions. In terms of mean arterial blood pressure and norepinephrine requirements there were no differences to observe between the study groups. pH and serum HCO_3_^-^ concentration decreased slightly but significantly only with Ringer’s lactate. In addition, the acetate-based solution kept the plasma effective strong ion difference more stable than Ringer’s lactate.

**Conclusions:**

Both of the solutions provided hemodynamic stability. Concerning consistency of acid base parameters none of the solutions seemed to be inferior, either. Whether the slight advantages observed for the acetate-buffered solution in terms of stability of pH and plasma HCO_3_^-^ are clinically relevant, needs to be investigated in a larger randomized controlled trial.

## Background

In the fields of surgery and intensive care, hyperchloremic acidosis is a well-known problem in patients receiving large amounts of standard crystalloids, especially 0.9% sodium chloride solutions. A series of investigations has emphasized the disadvantageous effects of hyperchloremic acidosis on various organ systems, for example, hemodynamics, NO-production, renal blood circulation, urinary output or hemostasis [1-4]. Balanced crystalloids, whose composition prevents hyperchloremia, are increasingly accepted and likely to be ‘state of the art’ in the near future [3,5-7]. Balanced hydroxyethyl-starch solutions are also available today. All of these preparations are characterized by the presence of metabolizable organic anions such as lactate, acetate or malate and contain physiological electrolyte concentrations. Theoretically, balanced solutions which possess a strong ion difference (SID) of around 24 mmol/L after metabolization (for example, Ringer’s lactate) are supposed to have no influence on blood pH even after intravenous infusion of several liters [8]. However, balanced crystalloids containing lactate have some disadvantages as well [9]. Their metabolism depends on liver function and does require increased oxygen consumption. Acetated solutions do not display these shortcomings, but previous studies in patients undergoing hemodialysis have demonstrated that their administration may lead to hemodynamic instability and hypotension [10].

Despite their future perspective, to date the routine use of balanced crystalloids is still infrequent in anesthesia. Large randomized, controlled clinical trials (RCT’s) investigating possible advantages as well as adverse effects of balanced crystalloids for routine use in the operating theater are sparse. The aim of the current study was to provide a preliminary comparison of two differently buffered balanced infusion solutions by reviewing data obtained from two established clinical protocols and,in addition, to test the feasibility of a future large RCT. The main target of interest was to investigate the influence of a solution containing a plasma-like SID (acetate buffered, SID = 36.8 mmol/L) and a solution with a comparably low SID (lactate buffered, SID = 28 mmol/L) on the variables of Stewart’s acid–base approach including the effective strong ion difference (SIDe). The impact of the acetate-based infusion solution on hemodynamic stability was also studied. In order to guide a future RCT, primary and secondary variables of interest were determined and sample size estimation was performed.

## Methods

In 2009 Ringer’s lactate (B. Braun Melsungen, Melsungen, Germany) was replaced by Ionosteril^TM^ (Fresenius Medical Care, Schweinfurt, Germany), an acetate-based infusion solution (ABIS), as a standard intraoperative infusion solution in our institution. In a before and after analysis we studied acid–base balance and hemodynamics in 24 women without obvious cardiac, pulmonary or renal diseases (classified as American Society of Anesthesiologists physical status I or II) who underwent major, open gynecologic cancer surgery. The study comprised two arms: the first consisted of patients who had received solely Ringer’s lactate (RL-group, n = 12) and the second of women who were treated only with the ABIS (ABIS-group, n = 12). The composition of both infusion solutions is listed in Table 1. Patients suffering from pre-existing acid–base disorders and patients who required blood transfusions, plasma products or colloids during the study period were not included in the investigation. A retrospective chart review was performed for patients in both arms. The mean intergroup difference of changes in SIDe between baseline values and the end of the study period was defined as the primary variable of interest. Changes in arterial plasma pH and noradrenalin requirements during general anesthesia were considered as secondary variables. The target parameters of the study were obtained from intraoperative routine blood samples, taken from arterial lines in patients requiring postoperative intensive care management (see Table 2). Because no additional blood sampling was necessary and the study was designed to evaluate two different versions of an established infusion regimen, the Ethics Committee of the University of Munich waived the need for patient’s informed consent for this pilot trial.

**Table 1:** Composition of study solutions

		**Ringer’s lactate**	**ABIS**
	mmol/L		
[Na^+^]		131	137
[Cl^-^]		112	110
[K^+^]		5.4	4
[Mg^++^]		0	1.25
[Ca^++^]		1.8	1.65
[Lactate]		28	0
[Acetate]		0	36.8
Strong ion difference		28	36.8
	mosmol/L		
Osmolarity (calculated)		276	291
Osmolality (*in vivo*)		256	270

[], serum concentration of the ion between the brackets; ABIS, acetate-based infusion solution.

**Table 2:** List of variables

	**Measurement at:**		**Measurement at:**
**Hemodynamics**		**Acid Base Balance**	
Noradrenalin requirement (mg/h)	BL – T120	Anion Gap (mmol/L)	BL – T120
Heart rate (min-1)	BL – T120	HCO3- (mmol/L)	BL – T120
ABP systolic (mmHg)	BL – T120	BE (mmol/L)	BL – T120
ABP diastolic (mmHg)	BL – T120	pH	BL – T120
Central venous pressure (mmHg)	BL – T120	paCO2 (mmHg)	BL – T120
Blood loss (mL)	BL – T120		
Hemoglobin (g/dL)	BL – T120	Stewart Acid Base Approach [A-] (mmol/L)	BL – T120
Hematocrit (%)	BL – T120	Effective Strong Ion Difference (mmol/L)	BL – T120
Urine quantity (mL)	BL – T120	Strong Ion Gap (mmol/L)	BL – T120
Administered amount of RL/ABIS (mL)	BL – T120	[Albumin] (g/L)	BL – T120
		[Serum-Lactate] (mmol/L)	BL – T120
Serum-Electrolytes([Na+][Cl-] [K+] [Mg++] [Ca++])	BL – T120	[A-]: = Serum-concentration of week acids	BL – T120

ABIS, acetate-based infusion solution; ABP, arterial blood pressure; BE, base excess; BL, baseline; [], serum concentration of the variable between the brackets.

General anesthesia was induced with intravenous propofol, sufentanil, and cis-atracurium. After tracheal intubation, anesthesia was maintained with propofol (6 to 10 mg/kg/hour) and additional doses of sufentanil and cis-atracurium as appropriate. Mechanical ventilation was performed to maintain an adequate oxygenation and to preserve normocapnia. Intraoperative monitoring included end-tidal P_a_CO_2_, electrocardiography, central venous pressure, arterial blood pressure, pulseoximetry and esophageal temperature. During the operative period, the patient’s temperature was kept constant in any case. Urinary output as well as the blood loss during the time course of the investigation was noted. In order to obtain blood pH, base excess (BE), HCO_3_^-^ and P_a_CO_2_, a standard blood gas analyzer (Bayer Rapidlab 1265, Bayer Healthcare LLC, East Walpole, MA, USA) was used. Serum electrolyte and lactate concentrations, as well as hemoglobin, hematocrit and albumin concentrations were assessed in the central laboratory of our institution. Depending on the results of the laboratory measurements the following variables were calculated:

· [PO_4_^3-^] ionized (Pi) (mmol/L): = [PO_4_^3-^] (mg/dL) · 0.3229

· Anion gap (mmol/L): = [Na^+^] – [Cl^-^] – [HCO_3_^-^]

· Effective Strong Ion Difference (SID_e_) (mmol/L): = [A-] + [HCO_3_^-^ = [A-] + (0.0304 · PCO_2_ · 10^(pH-6.1)^) according to Figge [11,12]

· Week acids serum-concentration [A^-^ (mmol/L): = [Albumin · (0.123 · pH – 0.631)] + [Pi · (0.309 · pH - 0.469)] according to Figge [11,12]

· Strong Ion Gap (SIG) (mmol/L): = [Na^+^ + [K^+^ + 2[Ca^++^ + 2[Mg^++^ – [Cl^-^–[Lac^-^–[A^-^–[HCO_3_^-^ according to Stewart [13]

### Hemodynamics

In accordance with our institution’s clinical practice a mean arterial pressure (MAP) of at least 80 mmHg was regarded as necessary during general anesthesia. If volume replacement with the study solutions alone (up to 30 ml/kg/hour) was not sufficient to maintain the necessary level of MAP norepinephrine was administered in increasing doses of 0.1 mg/hour as required until the target value was achieved.

### Statistical analysis

Mean, standard deviation of the mean (parametric values), median and range (non-parametric data) were calculated for each target parameter. The Shapiro-Wilk test was used to test for normality. Because most of the data were normally distributed, they are presented as mean ± standard deviation. We used a repeated measurement analysis of variances (RM-ANOVA) followed by a Student-Newman-Keuls test (with data normally distributed) or a Friedman test (with data not normally distributed) to describe changes of measurement parameters within a group during the course of time (three points of measurement). In order to compare differences between the groups an ANOVA was followed by a Student-Newmann-Keuls test (see above). If data were not normally distributed a one-way ANOVA on ranks (Kruskal-Wallis test) was followed by Dunn’s test. For all determinations a type I error protection of *P* <0.05 was considered significant. Statistical analysis was performed using Sigma Stat Software Version 3.1 (RockWare Inc. Golden, Colorado, USA) and Microsoft Excel Version 2003 (Microsoft Deutschland GmbH, Unterschleißheim, Germany). All electrolyte concentrations are expressed as mmolLl.

## Results

We did not notice significant differences concerning patients’ demographic data (mean age: RL-group, 64 ± 8; ABIS-group, 61 ± 10 years; mean body mass index: RL-group, 24.8 ± 2.6; ABIS-group, 24.5 ± 3.5; mean body weight: RL-group, 68.6 ± 6.0 kg; ABIS-group, 66.4 ± 14.0 kg) (*P* > 0.05 for all comparisons). All women underwent surgery for ovarian cancer including open radical hysterectomy, adnexal surgery and para-aortal as well as pelvic lymph node dissection. Twenty-four patients, who *a priori* fulfilled the inclusion criteria, were enrolled in this study (12 per group). Protocol violations were not observed and subsequently there was no need to retrospectively exclude patients from the trial. The mean intergroup difference of changes in SIDe between BL and T120 proved to be 2.08 ± 2.9 mmol/L, indicating a strong effect (d = 0.91) according to Cohen [14]. Thus, assuming an effect size of 2 mmol/L, a future RCT should include at least 35 patients in each study group, given a type II error protection of β > 0.8.

### Acid–base balance and Stewart approach

Parameters contributing to the ‘classic’ acid–base balance (see Table 3) did not differ significantly between groups at any time point of measurement. Intergroup comparisons concerning parameters relevant for Stewart’s acid–base approach, especially the SIDe, showed no significant differences, either. However, values noticed for the RL-group showed small but significant intragroup changes during the time course, especially concerning pH and [HCO_3_^-^]. In the ABIS-group, these values did not change. In addition, relevant intragroup reductions were observed in SID_e_ and [A^-^] for RL and ABIS. Albumin serum concentrations were diminished to approximately 80% of their basic values in both groups.

**Table 3:** Acid–base parameters

		**BL**		**T60**		**T120**		**ІΔ120І**
		**Mean ± SD**	**95% CI**	**Mean ± SD**	**95% CI**	**Mean ± SD**	**95% CI**	**Mean ± SD**
[HCO_3_^-^]	RL*	24.65 ± 2.69	(22.94-26.36)	23.86 ± 2.86	(22.04-25.68)	23.41 ± 3.62	(21.11-25.71)	1.24 ± 1.72
	ABIS	25.09 ± 2.52	(23.49-26.69)	25.19 ± 1.85	(24.01-26.37)	24.53 ± 2.31	(23.06-26.00)	0.56 ± 1.79
BE	RL*	0.38 ± 2.89	(−1.46-2.22)	−1.11 ± 3.3	(−3.21-0.99)	−1.63 ± 3.63	(−3.94-0.68)	2.0 ± 1.87
	ABIS	0.48 ± 2.63	(−1.19-2.15)	0.45 ± 1.99	(−0.81-1.71)	−0.07 ± 2.51	(−1.66-1.52)	0.54 ± 1.46
pH	RL*	7.42 ± 0.05	(7.39-7.45)	7.39 ± 0.04	(7.36-7.42)	7.37 ± 0.03	(7.35-7.39)	0.05 ± 0.02
	ABIS	7.41 ± 0.03	(7.39-7.43)	7.42 ± 0.04	(7.39-7.45)	7.39 ± 0.04	(7.36-7.42)	0.03 ± 0.03
p_a_CO_2_ (mmHg)	RL	39.56 ± 1.5	(38.61-40.51)	39.58 ± 1.46	(38.65-40.51)	40.73 ± 3.04	(38.80-42.66)	1.16 ± 2.69
	ABIS	40.35 ± 2.77	(38.59-42.11)	40.55 ± 2.85	(38.74-42.36)	40.95 ± 1.87	(39.76-42.14)	0.6 ± 2.40
[A^-^]	RL*	12.51 ± 1.2	(11.75-13.27)	10.52 ± 1.05	(9.85-11.19)	9.81 ± 2.0	(8.54-11.08)	2.69 ± 1.70
	ABIS*	12.80 ± 2.35	(11.31-14.29)	10.12 ± 1.39	(9.24-11.00)	10.12 ± 1.82	(8.96-11.28)	2.67 ± 2.40
SID_e_	RL*	37.90 ± 3.75	(35.52-40.28)	34.34 ± 3.19	(32.31-36.37)	33.20 ± 4.81	(30.16-36.28)	4.71 ± 2.71
	ABIS*	37.45 ± 3.00	(35.54-39.36)	35.34 ± 2.50	(33.75-36.93)	34.82 ± 4.00	(32.28-37.36)	2.63 ± 1.75
SIG	RL*	4.95 ± 3.38	(2.80-7.10)	1.67 ± 3.03	(−0.26-3.60)	0.73 ± 4.43	(−2.08-3.54)	4.21 ± 2.93
	ABIS°	2.98 ± 2.31	(1.51-4.45)	1.56 ± 1.0	(0.92-2.20)	2.44 ± 1.99	(1.18-3.70)	0.54 ± 2.76
Albumin[g/l]	RL*	37.0 ± 4.31	(34.26-39.74)	30.58 ± 3.68	(28.24-32.92)	28.41 ± 6.91	(24.02-32.80)	8.60 ± 5.71
	ABIS*	35.82 ± 7.70	(30.93-40.71)	29.18 ± 4.21	(26.51-31.85)	29.10 ± 5.58	(25.55-32.65)	6.72 ± 8.45

All values except p_a_CO_2_, albumin and pH are given in mmol/L. ABIS, acetate-based infusion solution; BE, base excess; BL, baseline measurement; RL, Ringer’s lactate; SD, standard deviation; SIDe, effective strong ion difference; SIG, strong ion gap; T60, T120, measurement at 60 ± 10 and 120 ± 10 minutes; 95% CI, 95% confidence interval of means; І Δ120 І, absolute value of the difference between T120 and baseline measurements. *, Significant intragroup changes over time (*P* < 0.05) between BL and T120; °, Significant intragroup changes over time (*P* < 0.05) between BL and T60.

### Hemodynamics

The overall mean blood loss during the time course was 763 ± 427 ml; it was 655 ± 460 ml in the RL-group and 870 ± 378 ml in the ABIS-group (*P* > 0.05). The total amount of the infused study solutions was 4,066 ± 308 ml for RL and 4,041 ± 572 ml for the ABIS (*P* > 0.05). Norepinephrine requirements increased constantly during the study period, but differences between groups did not reach significance. Nevertheless, a slight but significant reduction in CVP could be observed in the RL-group. There were no differences in urine output. Further parameters listed under ‘Hemodynamics’ in Table 4 were not significantly different between groups.

**Table 4:** Hemodynamics

		**BL**		**T60**		**T120**		**ІΔ120І**
		**Mean ± SD**	**95% CI**	**Mean ± SD**	**95% CI**	**Mean ± SD**	**95% CI**	**Mean ± SD**
Norepinephrine requirements (mg/hour)	RL*	0.1 ± 0.1	(0.04-0.16)	0.3 ± 0.2	(0.17-0.43)	0.4 ± 0.3	(0.21-0.59)	0.3 ± 0.3
	ABIS*	0.1 ± 0.1	(0.04-0.16)	0.3 ± 0.2	(0.17-0.43)	0.5 ± 0.4	(0.25-0.75)	0.41 ± 0.4
Urine-Quantity (ml)	RL*	8.3 ± 19.4	(−4.03-20.63)	210.8 ± 165.2	(105.84-315.76)	630.8 ± 422.2	(362.5-899.0)	622.5 ± 404.4
	ABIS*	15.8 ± 31.7	(−4.34-35.94)	216.6 ± 153.3	(119.20-314.00)	533.3 ± 286.2	(351.4-715.1)	517.5 ± 271.6
HR (min^-1^)	RL	55.2 ± 10.9	(48.27-62.13)	56.1 ± 11.8	(48.60-63.60)	58.7 ± 13.6	(50.06-67.34)	3.5 ± 5.3
	ABIS	55.0 ± 6.3	(51.00-59.00)	60.8 ± 10.4	(54.19-67.41)	59.9 ± 13.9	(51.07-68.73)	4.9 ± 10.0
MAP mmHg)	RL	86.4 ± 8.9	(80.75-92.05)	85.5 ± 11.7	(78.07-92.93)	84.4 ± 11.5	(77.09-91.71)	2.0 ± 11.2
	ABIS	88.9 ± 13.3	(80.45-97.35)	94.1 ± 15.7	(84.12-104.08)	91.3 ± 15.0	(81.77-100.83)	2.4 ± 14.8
CVP (mmHg)	RL*	11.2 ± 2.3	(9.74-12.66)	10.7 ± 3.5	(8.48-12.92)	8.8 ± 3.5	(6.58-11.02)	2.4 ± 3.4
	ABIS	10.9 ± 3.2	(8.87-12.93)	12.7 ± 5.3	(9.33-16.07)	11.4 ± 4.4	(8.60-14.20)	0.57 ± 5.4

ABIS, acetate-based infusion solution; BL, baseline measurement; CVP, central venous pressure; HR, heart rate; MAP, mean arterial pressure; RL, Ringer’s lactate; SD, standard deviation; T60, T120, measurement at 60 ± 10 and 120 ± 10 minutes; 95% CI, 95% confidence interval of means; І Δ120 І, absolute value of the difference between T120 and baseline measurements; *, significant intragroup changes over time (*P* < 0.05) between BL and T120.

### Electrolytes

Serum electrolytes were measured mainly to calculate the parameters of the Stewart approach. Except for [lactate^-^] and [Mg^++^] no significant differences between the study groups were observed (see Table 5). Significantly higher levels of [Mg^++^] could be found in the ABIS-group at T60 and T120. Serum lactate concentrations were significantly elevated for RL compared to ABIS at T60 and T120 (*P* < 0.05 for all comparisons). In addition, a significant increase in [Cl^-^] could be noticed during the time course in the RL-group (Table 5), but not in the ABIS-group.

**Table 5:** Serum electrolyte concentrations

		**BL**		**T60**		**T120**		**ІΔ120І**
		**Mean ± SD**	**95% CI**	**Mean ± SD**	**95% CI**	**Mean ± SD**	**95% CI**	**Mean ± SD**
[Na^+^]	RL*	137.3 ± 4.8	(134.2-140.4)	136.2 ± 4.7	(133.2-139.2)	135.8 ± 5.1	(132.5-139.0)	1.52 ± 1.65
	ABIS	138.9 ± 2.5	(137.3-140.5)	138.3 ± 2.3	(136.8-139.7)	138.5 ± 2.7	(136.8-140.2)	0.35 ± 2.9
[K^+^]	RL*	3.5 ± 0.2	(3.4-3.7)	3.6 ± 0.3	(3.40-3.79)	3.7 ± 0.5	(3.38-4.01)	0.26 ± 0.52
	ABIS*	3.5 ± 0.3	(3.31-3.70)	3.7 ± 0.3	(3.50-3.89)	3.7 ± 0.2	(3.57-3.82)	0.22 ± 0.31
[Cl^-^]	RL*	101.8 ± 5.4	(99.0-104.8)	105.8 ± 5.0	(102.6-109.0)	107.5 ± 4.6	(104.6-110.4)	5.60 ± 2.4
	ABIS	105.5 ± 3.3	(103.4-107.6)	108.4 ± 2.5	(106.8-110.0)	108.6 ± 2.2	(107.2-110.0)	3.08 ± 1.46
[Mg^2+^]	RL*	0.77 ± 0.1	(0.71-0.82)	0.69 ± 0.1#	(0.62-0.75)	0.66 ± 0.1#	(0.59-0.72)	0.11 ± 0.12
	ABIS	0.83 ± 0.06	(0.79-0.87)	0.85 ± 0.04#	(0.82-0.87)	0.87 ± 0.04#	(0.84-0.89)	0.04 ± 0.06
[Ca^2+^]	RL	1.16 ± 0.04	(1.13-1.19)	1.16 ± 0.04	(1.13-1.18)	1.16 ± 0.02	(1.14-1.17)	0.00 ± 0.03
	ABIS	1.17 ± 0.03	(1.15-1.19)	1.16 ± 0.03	(1.14-1.18)	1.16 ± 0.03	(1.14-1.17)	0.01 ± 0.02
[Lac^-^]	RL*	0.8 ± 0.3	(0.66-1.07)	1.6 ± 0.6#	(1.21-1.98)	1.9 ± 0.7#	(1.45-2.34)	0.94 ± 0.69
	ABIS	0.9 ± 0.4	(0.64-1.15)	0.8 ± 0.3#	(0.60-1.0)	0.8 ± 0.2#	(0.67-0.92)	0.12 ± 0.22
[PO_4_^3-^]	RL	3.5 ± 0.4	(3.21-3.78)	3.4 ± 0.4	(3.14-3.65)	3.3 ± 0.4	(3.04-3.55)	0.19 ± 0.32
(mg/dL)	ABIS	3.4 ± 0.5	(3.08-3.719	3.3 ± 0.5	(3.00-3.60)	3.4 ± 0.6	(3.02-3.78)	0.01 ± 0.28

All values except [PO_4_^3-^] are given in mmol/L. ABIS, acetate-based infusion solution; BL, baseline measurement; RL, Ringer’s lactate; SD, standard deviation; T60, T120, measurement at 60 ± 10 and 120 ± 10 minutes; 95% CI, 95% confidence interval of means; І Δ120 І, absolute value of the difference between T120 and baseline measurements; *, significant intragroup changes over time (*P* <0.05) between BL and T120; #, significant differences between groups at the referring time points of measurement (*P* < 0.05).

## Discussion

The current investigation was designed to compare two currently available balanced infusion solutions in a clinical setting. One solution contained lactate, the other acetate as a metabolizable anion. Main areas of interest were the influence of these solutions on acid–base balance and hemodynamic stability.

### Acid–base balance

**Figure 1:**
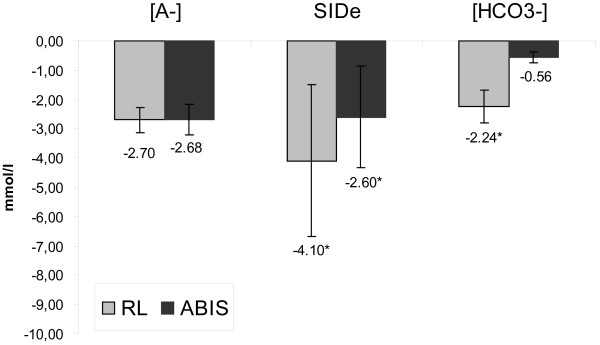
**Reduction of [A**^**-**^**], SID**_**e**_**and [HCO**_**3**_^**-**^**] during the time course (BL – T120).** ABIS, acetate-based infusion solution; BL, baseline; RL, Ringer’s lactate; T120, measurement at 120 ± minutes; SID_e,_ effective strong ion difference; *, significant intragroup changes over time (*P* <0.05) between BL and T120.

On first sight, the acid–base parameters pH, BE and [HCO_3_^-^ remained remarkably constant during the time course. This observation corresponds to the results of previous investigators [4,8]. However, it was evident that the ABIS had less influence on the ‘classic’ acid–base parameters than RL. pH, [HCO_3_^-^ and BE did not change from BL to T120 in the ABIS-group, whereas a small but significant reduction of pH values and [HCO_3_^-^ could be observed in the RL-group. Concerning the parameters of Stewart’s acid–base model, we noticed a relevant reduction in serum albumin concentrations, which is easily explained by the diluting effects of the albumin-free crystalloid solutions. This reduction was accompanied by a corresponding decrease in [A^-^ (Δ BL-T120: RL-group, 2.70 mmol/L; ABIS-group, 2.68 mmol/L). In addition, the SID_e_ declined as well. In contrast to [A^-^ a small difference in the quantity of the decline could be observed with SID_e_ (Δ BL-T120: RL-group, 4.7 mmol/l; ABIS-group, 2.6 mmol/L; *P* > 0.05). Although the difference in SID_e_-reduction was not significant between groups, one can speculate that the pronounced decrease of effective SID in the RL-group (which should lead to an acidosis according to the Stewart approach) was only incompletely neutralized by the decrease in [A^-^ (which should lead to an alkalosis). This might explain the small but significant pH reduction that could be observed in the RL-group (see Figure 1).

This finding is surprising, because it does not correspond to Morgan’s theory of the ‘ideal SID’ [7]. According to Morgan *et al*. only infusion solutions with a SID of around 24 mmol/L do not influence blood pH. As a consequence one should have expected RL (SID 28 mmol/L) rather than ABIS (SID 36.8 mmol/L) to guarantee pH stability and ABIS to cause a mild alkalosis. Nevertheless, in the current study ABIS, whose SID nearly approximates normal plasma SID_e_ (39 ± 4 mmol/L), provided better pH stability [15]. We have no explanation for the discrepancy concerning Morgan’s theory. One can only speculate that perhaps infusion rate and/or metabolic turnover of the metabolizable anions may be important in this situation. In addition, according to Wooten’s multi-compartmental model, not only extracellular, but also intracellular effects, and fluid redistribution between compartments may have influenced acid–base chemistry [16].

One possible limitation of the current study was that some of the values (especially the SID) had to be calculated from directly measured parameters. With differences between and within groups being very small, a propagation of errors could have influenced group comparisons. However, modern blood gas analyzers, as used in the current study, are able to measure acid–base variables extremely precisely (pH bias <0.005, PaCO_2_ bias < 1.2 mmHg) [17]. In addition, measurement errors concerning the components of SID should occur similarly in all patients. Therefore, differences between groups should not be affected.

In summary, there were no significant intergroup differences to observe. Compared to RL, ABIS showed less influence on the ‘classic’ acid–base parameters (pH, BE, [HCO_3_^-^) and the solution did not provoke a clinically relevant change in plasma SID_e_. These results are in accordance with a recently published investigation on trauma patients requiring intensive care treatment with regard to pH stability provided by acetated solutions [18]. The clinical relevance of the relatively small intragroup changes in this study may be a matter of discussion. Scientific research on hyperchloremic acidosis has revealed that even small changes in pH can have a negative impact on various organ systems [1,2,4,19]. In addition, fluid requirements during major surgery may exceed by far the amount of four liters of crystalloids administered during the current study. Thus, small (but significant) differences concerning acid–base stability of applied crystalloids may gain importance successively if increasing amounts of fluids are administered.

### Hemodynamic values

Hemodialysis with acetate-containing solutions can be the cause of severe hemodynamic instability because of increased nitric oxide synthesis and enhanced arrhythmias [20-22]. Even the small quantity of acetate present in various dialysis fluids (usually 35 mmol/L) exposes a patient's blood to an acetate concentration 10 to 40 times the physiological level (50 to 100 μmol/L) [23-25]. Fournier *et al*. observed that, according to the nature of hemodialysis (counter current process), the concentration of plasma acetate increased from 0.02 mmol/L to 0.22 mmol/L in three hours of hemodialysis [25]. To date, it is unknown whether the administration of acetated infusion solutions can provoke substantial rises in plasma acetate levels as well. However, one can speculate that while (slowly) infusing acetate-containing solutions, a considerably greater amount of the substance (compared to hemodialysis) will be metabolized. This should lead to relatively low serum concentrations.

This assumption is supported by two different observations: Firstly, no significant differences could be observed in terms of hemodynamic values between the study groups. Secondly, the SIG in the ABIS-group did not change significantly during the time course. According to Stewart’s approach, an increase in plasma acetate must lead to an increase in SIG [13]. This hypothesis has been confirmed by Bruegger *et al*. in a hemorrhagic shock canine model: A 2.2 mmol/L rise in acetate plasma levels caused an identical (absolute) increase in SIG [26]. As a consequence, it is unlikely that relevant amounts of acetate accumulated in the circulation in the ABIS-group. However, hemodynamic stability could only be maintained by administering increasing amounts of norepinephrine (*P* < 0.05 from BL to T120 in both of the groups). There are several possible explanations: Firstly, the range of blood loss was relatively broad (from 50 ml up to 1,500 ml). Despite fluid replacement, which, according to the literature, should have been adequate, these fluid losses were obviously not sufficiently counterbalanced in some of the patients, especially in the ABIS-group [27,28]. It seems probable that some patients developed a mild hypovolemia during the time course of the study. This assumption is supported by a decreasing CVP in the ABIS group, in which blood loss tended to be more severe than in the RL-group. Secondly, one has to take into account the mild vasodilatory effect of the used narcotics (propofol) that may have contributed to norepinephrine requirements. Thus, when considering only the current, preliminary data it seems that both solutions ‘behaved like normal crystalloids’ without substance-specific hemodynamic effects. However, it will be interesting to investigate if small intergroup differences observed for CVP or MAP which, nevertheless, are of clinical importance, will gain significance in a larger RCT.

### Additional findings

Slightly elevated lactate levels were observed in the RL-group. These increases were only moderate (but significant) and hardly exceeded our institutions’ range of normal values (0.5 to 1.5 mmol/L). In contrast to ABIS [Mg^++^] significantly decreased in the RL-group. Because RL does not contain magnesium, this can best be explained by a simple dilution effect. We consider these changes to be of minor clinical importance. However, for patients suffering from liver or metabolic diseases or cardiac arrhythmias ABIS might be the preferable infusion solution.

### Feasibility of a future RCT

The current study showed that a larger RCT is feasible. The preliminary sample size estimation did not include the possibility of protocol violations or dropouts. Therefore, at least 35 patients will have to be enrolled in each group. With regard to the consistency of the study population in terms of co-factors of surgery and demographic data it is likely that a large RCT will provide reliable and reproducible results, despite the small intergroup differences in primary and secondary values of interest we observed.

## Conclusions

In the current preliminary comparison Ringer’s lactate as well as ABIS proved to be suitable for fluid replacement during abdominal surgery. Hemodynamic stability remained unaffected by both of the solutions. Concerning consistency of acid base parameters none of the solutions seemed to be clearly inferior, either. ABIS had a smaller effect on pH, BE, [HCO3-] and SIDe changes than lactated Ringer, but whether these differences will turn out to be of clinical relevance has to be investigated in a larger RCT. With regard to SIDe and [A-] neither ABIS nor Ringer’s lactate seem to be ‘ideally balanced’, because both of these values decreased dose dependently in both groups. A future RCT might provide important data concerning the on-going search for an ideal balanced solution.

## Abbreviations

[]: serum-concentration of the ion between the brackets; [A-]: serum-concentration of week acids; ABIS: acetate-based infusion solution; ABP: arterial blood pressure; BE: base excess; CVP: central venous pressure; HR: heart rate; MAP: mean arterial pressure; Pi: [PO_4_^3-^] ionized; RCT: randomized controlled trial; RL: Ringer’s lactate; SID: strong ion difference; SIDe: effective strong ion difference; SIG: strong ion gap.

## Competing interests

This study was performed using research funding provided by Fresenius Medical Care (Fresenius Medical Care Deutschland GmbH, Hafenstr. 9, 97424 Schweinfurt, Bayern, Germany). Fresenius Medical Care did not have any influence on study design or manuscript approval. Klaus Hofmann-Kiefer, Matthias Jacob and Markus Rehm have lectured for Baxter Deutschland GmbH (Unterschleißheim, Germany), Fresenius Kabi Deutschland GmbH (Bad Homburg, Germany), B Braun, Melsungen AG (Melsungen, Germany), Serumwerk Bernburg AG (Bernburg, Germany) and CSL Behring GmbH (Marburg, Germany). In addition, they received unrestricted research grants from Serumwerk Bernburg AG (Bernburg, Germany), CSL Behring GmbH (Marburg, Germany) and Fresenius Kabi Deutschland GmbH (Bad Homburg, Germany). All authors declare they have no competing interests.

## Authors’ contributions

KH-K made substantial contributions to the development of the study design, wrote the manuscript and performed the statistics. DC participated in data acquisition and analysis and critically revised the manuscript. TK and MP participated in data acquisition and patient coordination. MJ and PC critically revised and helped to draft the manuscript. MR participated substantially in the design and coordination of the study and gave final approval of the version to be published. All authors read and approved the final manuscript.

## References

[B1] Kellum JA Song M Venkataraman R Effects of hyperchloremic acidosis on arterial pressure and circulating inflammatory molecules in experimental sepsis Chest 2004 125 243 248 10.1378/chest.125.1.243 14718447

[B2] Kellum JA Song M Li J Lactic and hydrochloric acids induce different patterns of inflammatory response in LPS-stimulated RAW 264.7 cells Am J Physiol Regul Integr Comp Physiol 2004 286 R686 R692 10.1152/ajpregu.00564.2003 14695114

[B3] Kellum JA Metabolic acidosis in the critically ill: lessons from physical chemistry Kidney Int Suppl 1998 66 S81 S86 9573580

[B4] Wilkes NJ Woolf R Mutch M Mallett SV Peachey T Stephens R Mythen MG The effects of balanced versus saline-based hetastarch and crystalloid solutions on acid–base and electrolyte status and gastric mucosal perfusion in elderly surgical patients Anesth Analg 2001 93 811 816 10.1097/00000539-200110000-00003 11574338

[B5] Kellum JA Clinical review: reunification of acid–base physiology Crit Care 2005 9 500 507 10.1186/cc3789 16277739 PMC1297616

[B6] Kellum JA Saline-induced hyperchloremic metabolic acidosis Crit Care Med 2002 30 259 261 10.1097/00003246-200201000-00046 11902280

[B7] Morgan TJ Venkatesh B Designing ‘balanced’ crystalloids Crit Care Resusc 2003 5 284 291 16563119

[B8] Scheingraber S Rehm M Sehmisch C Finsterer U Rapid saline infusion produces hyperchloremic acidosis in patients undergoing gynecologic surgery Anesthesiology 1999 90 1265 1270 10.1097/00000542-199905000-00007 10319771

[B9] Guidet B Soni N Della RG Kozek S Vallet B Annane D James M A balanced view of balanced solutions Crit Care 2010 14 325 10.1186/cc9230 21067552 PMC3219243

[B10] Bruges M Barata JD Oliveira C Furstenau C Gomes EM Simoes J Hemodialysis with bicarbonate 30 mEq/l versus 34 mEq/l and acetate: better hemodynamic tolerance and electrolyte and acid–base homeostasis Acta Med Port 1994 7 165 170 8209702

[B11] Figge J Mydosh T Fencl V Serum proteins and acid–base equilibria: a follow-up J Lab Clin Med 1992 120 713 719 1431499

[B12] Figge J Rossing TH Fencl V The role of serum proteins in acid–base equilibria J Lab Clin Med 1991 117 453 467 2045713

[B13] Stewart PA Modern quantitative acid–base chemistry Can J Physiol Pharmacol 1983 61 1444 1461 10.1139/y83-207 6423247

[B14] Cohen J Statistical Power Analysis for the Behavioral Sciences 1988 2 Hillsdale: Lawrence Erlbaum Associates 76 84

[B15] Hofmann-Kiefer K Conzen P Rehm M Buchardi H, Larsen R, Kuhlen KW, Schölmerich J, Jauch KW Säure-Basen Status. In Die Intensivmedizin. 10th edition 2008 Heidelberg: Springer 744 753

[B16] Wooten EW Calculation of physiological acid–base parameters in multicompartment systems with application to human blood J Appl Physiol 2003 95 2333 2344 12923118 10.1152/japplphysiol.00560.2003

[B17] Wahr JA Lau W Tremper KK Hallock L Smith K Accuracy and precision of a new, portable, handheld blood gas analyzer, the IRMA 1 J Clin Monit 1996 12 317 324 10.1007/BF02221753 8863112

[B18] McCague A Dermendjieva M Hutchinson R Wong DT Dao N Sodium acetate infusion in critically ill trauma patients for hyperchloremic acidosis Scand J Trauma Resusc Emerg Med 2011 19 24 10.1186/1757-7241-19-24 21486493 PMC3087685

[B19] Kellum JA Fluid resuscitation and hyperchloremic acidosis in experimental sepsis: improved short-term survival and acid–base balance with Hextend compared with saline Crit Care Med 2002 30 300 305 10.1097/00003246-200202000-00006 11889298

[B20] Santoro A Guarnieri F Ferramosca E Grandi F Acetate-free biofiltration. Contrib Nephrol 2007 158 138 152 10.1159/000107244 17684352

[B21] Amore A Cirina P Mitola S Peruzzi L Bonaudo R Gianoglio B Coppo R Acetate intolerance is mediated by enhanced synthesis of nitric oxide by endothelial cells J Am Soc Nephrol 1997 8 1431 1436 9294835 10.1681/ASN.V891431

[B22] Shiohira S Kikuchi K Yoshida T Tsukada M Nitta K Akiba T A case report of the effect of acetate-free biofiltration on arrhythmia in a hemodialysis patient Ther Apher Dial 2007 11 155 158 10.1111/j.1744-9987.2007.00428.x 17381538

[B23] Coll E Perez-Garcia R Rodriguez-Benitez P Ortega M Martinez MP Jofre R Lopez-Gomez JM Clinical and analytical changes in hemodialysis without acetate Nefrologia 2007 27 742 748 18336105

[B24] Bottger I Deuticke U Evertz-Prusse E Ross BD Wieland O On the behavior of the free acetate in the miniature pig. Acetate metabolism in the miniature pig Z Gesamte Exp Med 1968 145 346 352 10.1007/BF02044224 5675250

[B25] Fournier G Potier J Thebaud HE Majdalani G Ton-That H Man NK Substitution of acetic acid for hydrochloric acid in the bicarbonate buffered dialysate Artif Organs 1998 22 608 613 10.1046/j.1525-1594.1998.06205.x 9684700

[B26] Bruegger D Kemming GI Jacob M Meisner FG Wojtczyk CJ Packert KB Keipert PE Faithfull NS Habler OP Becker BF Rehm M Causes of metabolic acidosis in canine hemorrhagic shock: role of unmeasured ions Crit Care 2007 11 R130 10.1186/cc6200 18081930 PMC2246228

[B27] Chappell D Jacob M Hofmann-Kiefer K Conzen P Rehm M A rational approach to perioperative fluid management Anesthesiology 2008 109 723 740 10.1097/ALN.0b013e3181863117 18813052

[B28] Prough DS Bidani A Hyperchloremic metabolic acidosis is a predictable consequence of intraoperative infusion of 0.9% saline Anesthesiology 1999 90 1247 1249 10.1097/00000542-199905000-00003 10319767

